# Quorum sensing in oral multispecies biofilms: from interkingdom communication to therapeutic targeting

**DOI:** 10.3389/fmicb.2026.1957601

**Published:** 2026-09-07

**Authors:** Yeping Lu, Yichao Xia, Jie Zhang, Fangyong Zhu, Zelda Ziyi Zhao

**Affiliations:** 1 Department of Stomatology, Shanghai East Hospital, Tongji University School of Medicine, Shanghai, China; 2 Department of Stomatology, Affiliated Hospital of Jiangnan University, Wuxi, China

**Keywords:** microbial interaction, oral biofilm, periodontitis, quorum quenching, quorum sensing

## Abstract

Oral biofilms are polymicrobial ecosystems where quorum sensing (QS) governs community assembly, virulence and homeostasis. This narrative review synthesizes evidence on QS pathways in key periodontal and cariogenic pathogens, emphasizing interspecies synergy, cross-kingdom interactions, and disease progression. We surveyed English-language publications up to 2026 across PubMed, Web of Science, and Scopus. Our analysis reveals three major QS circuits: the widely conserved LuxS/AI-2 system facilitating cooperative and competitive behaviors, such as *Fusobacterium nucleatum*-red complex coaggregation and *Veillonella*-mediated inhibition; Gram-positive oligopeptide systems (e.g., CSP (competence-stimulating peptide) /ComDE in *Streptococcus mutans*) governing competence and bacteriocin production; and the specialized acyl-homoserine lactone (AHL/AI-1) system in Gram-negative species, which fine-tunes biofilm maturation, heme acquisition, and interkingdom signaling. Beyond these canonical pathways, recent evidence has revised key assumptions: AI-2 production in *Fusobacterium nucleatum* is subspecies-specific and uncoupled from QS, while LuxS in *Porphyromonas gingivalis* exerts AI-2-independent effects on host immune modulation. These findings suggest that QS-inhibitor strategies must account for subspecies-level heterogeneity and distinguish signaling effects to inform targeted design. Pathogenically, QS drives alveolar bone resorption, macrophage-mediated inflammation, and metabolic crosstalk. Therapeutically, diverse QS-inhibitors including natural polyphenols, synthetic small molecules, and AHL-lactonases have indicated promising antibiofilm efficacy *in vitro* and in animal models. However, clinical translation remains hampered by poor oral retention, safety uncertainties, and a lack of standardized endpoints. We conclude that QS represents a potential druggable vulnerability in oral biofilms, but future efforts must prioritize high-specificity inhibitors, combination regimens with conventional antibiotics, and rigorous *in vivo* validation to bridge the translational gap.

## 1 Introduction

The oral cavity harbors one of the most diverse microbial communities in the human body, with over 700 bacterial species organized into structurally organized biofilms adhered to dental surfaces, mucosa, and prosthetic materials. These biofilms are not random aggregates but highly coordinated societies where cell-density-dependent communication, termed quorum sensing (QS), directs collective behaviors including virulence factor secretion, nutrient acquisition, stress tolerance, and spatial organization (Azimi et al., 2020). QS operates through the production, release, and detection of signaling molecules termed autoinducers (AIs), enabling bacteria to synchronize gene expression once a critical population threshold is reached.

In Gram-negative oral pathogens, the LuxI/LuxR-type acyl-homoserine lactone (AHL, AI-1) system is a key regulator, while Gram-positive species frequently rely on post-translationally modified oligopeptides, known as autoinducing peptides (AIPs), detected by two-component sensory systems. Uniquely, the LuxS/AI-2 pathway, synthesized by a conserved enzyme LuxS, is broadly distributed. This characterization requires qualification, including the dual metabolic/signaling role of LuxS and subspecies-specific heterogeneity (Pereira et al., 2013). It is produced and recognized by both Gram-negative and Gram-positive bacteria, making it a central hub for community-level coordination (Elmanfi et al., 2018).

Dysregulation of QS-driven homeostasis leads to dysbiosis, a microbial imbalance that underpins the two most prevalent oral diseases: periodontitis and dental caries (Hashim et al., 2026; Shanker and Federle, 2017; Ramsundar et al., 2023a). Periodontitis arises from inflammation triggered by dysbiotic plaque communities enriched in keystone pathogens such as *Porphyromonas gingivalis* (*P. gingivalis*), *Treponema denticola* (*T. denticola*), and *Tannerella forsythia* (*T. forsythia*) (the ‘red complex’), whereas caries is driven by acidogenic and aciduric species, particularly *Streptococcus mutans* (*S. mutans*). Traditional antimicrobial strategies, including mechanical debridement and systemic or local antibiotics, often fail to fully eradicate biofilms and contribute to resistance selection, creating an urgent need for anti-virulence strategies that disarm pathogens without killing them. QS inhibitors (QSIs) offer such an alternative by disrupting bacterial communication, thereby attenuating virulence and biofilm resilience while imposing minimal selective pressure for resistance (Odularu et al., 2022; Breijyeh et al., 2020; Cui and Kim, 2024; Vinodhini and Kavitha, 2024; Carradori et al., 2020). This paradigm shift from bactericidal to anti-virulence strategies has been articulated as a fundamental reorientation in antimicrobial drug discovery (Omwenga and Awuor, 2024).

This narrative review aims to systematically dissect QS circuits in key oral pathogens and their role in interspecies and cross-kingdom dynamics; to consolidate the pathogenic significance of QS in driving tissue destruction; to critically evaluate the landscape of QSIs derived from natural and synthetic sources, including enzymatic quorum quenchers; and to identify translational bottlenecks and future research priorities. This narrative review was informed by a targeted literature search in PubMed, Web of Science, and Scopus up to 2026, using the keywords ‘quorum sensing’ OR ‘QS’ AND ‘oral microbiome.’ Consistent with the narrative review format, we did not perform a formal systematic search with predefined inclusion/exclusion criteria or quantitative synthesis. Rather, we applied a critical and selective approach to identify: (i) well-characterized periodontal and cariogenic pathogens; (ii) recent studies (particularly within the last five years) that revise established paradigms; and (iii) therapeutic strategies with demonstrable translational potential. Titles and abstracts were screened for relevance, and full texts were assessed based on these thematic priorities, with a deliberate emphasis on original research and high-quality reviews that inform interspecies interactions and therapeutic targeting.

## 2 QS circuits in key oral pathogens and community dynamics

### 2.1 Pathway-specific mechanisms in major species

#### 2.1.1 LuxS/AI-2 system: a widely conserved but functionally heterogeneous interspecies signal

Before examining LuxS/AI-2 in individual species, a conceptual distinction is essential. LuxS is a bifunctional enzyme: it catalyzes the final step in the activated methyl cycle (converting S-ribosylhomocysteine to homocysteine and 4,5-dihydroxy-2,3-pentanedione, which spontaneously cyclizes to AI-2) and is therefore required for both AI-2 production and methionine recycling. Consequently, *luxS* deletion mutants exhibit pleiotropic phenotypes that may arise from disruption of the activated methyl cycle rather than loss of QS signaling per se (Pereira et al., 2013). Thus, attributing a phenotype to AI-2-dependent QS requires demonstration that the phenotype is (i) density-dependent, (ii) restored by exogenous AI-2 (or its precursor DPD), and (iii) mediated through a cognate AI-2 receptor. Where these criteria are not met, effects should be interpreted as LuxS-dependent but not necessarily QS-dependent (Vendeville et al., 2005).

The AI-2 synthase LuxS is widely distributed among oral bacteria. In *P. gingivalis*, the LuxS open reading frame was initially identified via sequence homology, showing 30% identity and 50% similarity with known LuxS proteins, and subsequent functional studies suggested that LuxS regulates the cysteine proteases Arg-gingipain (Rgp) and Lys-gingipain (Kgp), which are critical for host tissue invasion and immune evasion (Frias et al., 2001; Burgess et al., 2002). Beyond proteases, AI-2 signaling in *P. gingivalis* modulates heme acquisition through the HmuY and HmuR system, with transcription of the *hmu* genes governed by the LuxR-family regulator PG1237 (Wu et al., 2009). Beyond AI-2-dependent regulation, LuxS in *P. gingivalis* also exerts AI-2-independent effects by modulating host inflammatory responses through the outer membrane protein PGN_0482. A *luxS* mutant showed impaired induction of IL-1β, IL-6, and MCP-1 in periodontal ligament fibroblasts, a phenotype restored by *trans*-complementation with LuxS but not by the AI-2 precursor DPD, indicating AI-2 independence. A *PGN_0482* deletion mutant phenocopied the *luxS* mutant, establishing that LuxS influences host–pathogen interactions through AI-2-independent regulation of this immunomodulatory surface protein (Scheres et al., 2015).

In *Fusobacterium nucleatum* (*F. nucleatum*), a pivotal “bridge” organism connecting early and late colonizers, AI-2 signaling dramatically enhances biofilm robustness. Partially purified *F. nucleatum* AI-2 upregulates mRNA synthesis of the adhesin FadA, and importantly, it induces expression of red-complex adhesion molecules including RgpA in *P. gingivalis*, Msp in *T. denticola*, and BspA in *T. forsythia*, thereby promoting coaggregation and synergistic pathogenicity (Jang et al., 2013). Furthermore, *F. nucleatum* AI-2 promotes RANKL-induced osteoclast differentiation in a dose-dependent manner, directly exacerbating alveolar bone loss in murine periodontitis models (Li et al., 2024). However, these observations warrant reconsideration in light of recent genomic and functional evidence. Bibek et al. systematically examined AI-2 production across all four *F. nucleatum* subspecies and found that it is restricted exclusively to FNA strains, as FNN and FNV completely lack *luxS* while FNP carries a disrupted homolog. AI-2 bioassays using the *Vibrio harveyi* BB170 reporter detected AI-2 only in FNA strains, and *luxS* deletion in FNA abolished AI-2 production but resulted in minimal transcriptional changes, with exogenous AI-2 failing to elicit strong responses in non-producing subspecies. Collectively, these findings suggest that AI-2 production in *F. nucleatum* is subspecies-specific and uncoupled from QS, fundamentally revising the prevailing assumption that AI-2 functions as a conserved interspecies signal in this species complex and suggesting that subspecies-specific metabolic traits may underlie its ecological adaptation and pathogenic potential (Bibek et al., 2026). These findings align with the broader recognition that LuxS has a primary metabolic role as part of the activated methyl cycle, and phenotypes observed in *luxS* mutants cannot be automatically attributed to loss of QS/AI-2 signaling (Vendeville et al., 2005; Rezzonico et al., 2012).

For *Aggregatibacter actinomycetemcomitans* (*A. actinomycetemcomitans*), the AI-2 system has been extensively characterized. Fong et al. first demonstrated that *A. actinomycetemcomitans* expresses a functional *luxS* homolog and secretes an AI-2-like signal that induces luminescence in the *Vibrio harveyi* BB170 reporter. Importantly, exogenous AI-2 upregulated leukotoxin expression approximately threefold and induced the iron transport gene *afuA* eightfold, suggesting that LuxS-dependent signaling modulates both virulence and iron acquisition (Fong et al., 2001). The cellular receptor for AI-2 was later identified as RbsB, a ribose binding protein related to *V. harveyi* LuxP. Purified RbsB inhibited AI-2-induced bioluminescence in a dose-dependent manner, and an *rbsB* mutant showed reduced growth under iron limitation and decreased expression of the ferric ion transporter *afuABC* (James et al., 2006). Furthermore, it also revealed that both *luxS* and *arcB* mutants exhibited similar phenotypes under aerobic iron-limiting conditions, with reduced expression of iron transport and storage genes including *afuA*, *fecBCDE*, and *ftnAB*, suggesting that LuxS and ArcB act in concert to control adaptation to iron limitation (Fong et al., 2003). Beyond these regulatory functions, it showed that AI-2-containing spent media from *A. actinomycetemcomitans* significantly reduced dual-species biofilm biomass with *Candida albicans* and *Streptococcus mutans*, without substantially affecting the survival rate of *S. mutans*, indicating that AI-2 can modulate cross-kingdom biofilm architecture rather than simply inhibiting growth (Bachtiar and Bachtiar, 2020). At the level of biofilm formation, inactivation of AI-2 receptors RbsB and LsrB nearly abolishes biofilm development (Shao et al., 2007). The QseBC two-component system, part of the AI-2 regulon, links signal detection to biofilm maturation and bone-resorptive virulence (Novak et al., 2010). In dual-species cultures with *Actinomyces odontolyticus* (*A. odontolyticus*) and its epibiotic parasite TM7x, AI-2 operons including *lsrB* and *luxS* are significantly upregulated, underscoring the role of AI-2 signaling in parasitic and epibiotic interactions (Bedree et al., 2018). Notably, in a primate model, while an *luxS* mutant of *A. actinomycetemcomitans* showed minimal fitness loss, compensatory AI-2 from the resident microbiota and upregulation of attachment genes including *flp* sustained its colonization, highlighting the redundancy of AI-2 sources in complex communities (Velusamy et al., 2017).

Among other species, *Eikenella corrodens* (*E. corrodens*), which is prevalent in advanced periodontitis, possesses a functional *luxS* ortholog; AI-2 production peaks in mid-log phase and declines in stationary phase, with LuxS facilitating biofilm maturation and detachment (Azakami et al., 2006; Karim et al., 2013a; Karim et al., 2013b). In *Enterococcus faecalis* (*E. faecalis*), a common isolate in apical periodontitis, *luxS* deletion increases biofilm formation and cell-surface hydrophobicity, indicating a negative regulatory role (He et al., 2016; Yang et al., 2018). Similarly, LuxS deficiency in *Capnocytophaga ochracea* (*C. ochracea*) reduces biofilm formation, while *Veillonella tobetsuensis* (*V. tobetsuensis*) produces an AI-2-like molecule (PPAI-2) that significantly inhibits *Streptococcus gordonii* (*S. gordonii*) biofilm, representing an example of antagonistic QS-mediated interference (Hosohama-Saito et al., 2016; Mashima and Nakazawa, 2017).

#### 2.1.2 Gram-positive oligopeptide systems (AIP and CSP)

While much attention has focused on AI-2, Gram-positive oral species employ oligopeptide-based QS. In *S. mutans*, the competence-stimulating peptide (CSP) pheromone, processed from ComC and sensed by the ComDE two-component system, regulates bacteriocin production, acid tolerance, genetic competence, and biofilm formation. Under specific stresses, high CSP concentrations induce a suicidal altruistic response in a subpopulation, potentially aiding survival of the community (Dufour and Lévesque, 2013). Importantly, CSP pathways are subject to cross-species interference: CSP from *Streptococcus pneumoniae* (*S. pneumoniae*) can activate the *S. mutans* ComDE system, inducing biofilm and bacteriocin production (Bernabè et al., 2022). Conversely, proteases from other streptococci such as *Streptococcus sanguinis* (*S. sanguinis*) and *Streptococcus mitis* (*S. mitis*) can inactivate *S. mutans* CSP, curbing its pathogenic potential (Ricomini Filho et al., 2019). The Rgg/SHP system in *S. mitis* regulates its own *shp* gene and cross-activates the Rgg/SHP system in *S. pneumoniae*, modulating surface polysaccharide synthesis during multispecies interactions (Junges et al., 2019). These findings indicate that Gram-positive QS networks are highly interwoven and subject to both synergy and competition.

#### 2.1.3 AHL (AI-1) system: a specialized intraspecies and interkingdom cue

Distinct from the broadly distributed AI-2, acyl-homoserine lactones, collectively referred to as AHLs or AI-1, primarily mediate intraspecies or closely related Gram-negative communication, serving as a dedicated signaling line for fine-tuned regulation.

In key periodontal pathogens, *P. gingivalis* exhibits a notable dual-system strategy. Beyond its LuxS/AI-2 circuitry, it produces trace amounts of *N*-3-oxo-octanoyl-L-homoserine lactone (OC8-HSL) in monospecific cultures (Muras et al., 2020b). More importantly, the LuxR-family transcriptional regulator PG_2094 is significantly upregulated in monospecies biofilms compared to planktonic cells, suggesting that the AI-1 pathway actively contributes to sessile growth and community establishment (Sánchez et al., 2019). Additionally, another LuxR-family member, PG1237, directly controls the expression of hemin-binding protein genes (*hmu*), thereby modulating heme acquisition and bacterial survival under iron-restricted conditions (Wu et al., 2009).

AHL-mediated interspecies and interkingdom interference is particularly evident among opportunistic pathogens. The discussion that follows draws on well-characterized model organisms including *Pseudomonas aeruginosa* (*P. aeruginosa*), *Acinetobacter baumannii* (*A. baumannii*), and *Staphylococcus aureus* (*S. aureus*) to illustrate fundamental AHL-mediated QS principles. While these species are not primary oral pathogens, they provide mechanistic insights into AHL signaling dynamics that are applicable to the oral biofilm context. Where findings from these model organisms are presented, we explicitly indicate their general relevance and avoid extrapolating them as direct evidence for periodontal pathogenesis. *P. aeruginosa* secretes AHL molecules such as *N*-dodecanoyl-L-homoserine lactone (C12-HSL), which not only modulate osteoblast apoptosis and differentiation via intracellular calcium signaling but also activate the host bitter receptor T2R38 expressed in tumor cells, leading to upregulation of multidrug resistance protein 1 (MDR1) (Guo et al., 2020; Gaida et al., 2016). This provides a novel mechanistic link between oral and opportunistic pathogens and systemic conditions including cancer drug resistance. Within the oral niche, exogenous addition of *N*-hexanoyl-L-Homoserine lactone (C6-HSL) to *in vitro* dental plaque biofilms significantly alters lactic acid production, protease activity, and bacterial community composition, suggesting that AHL-driven QS participates in plaque maturation and ecological dysbiosis (Muras et al., 2022).

Furthermore, AHL systems are subject to complex interspecies and cross-kingdom interactions that can be antagonistic, synergistic, or inhibitory. *S. aureus*, a significant Gram-positive pathogen in oral infections, responds to *N*-3-oxo-dodecanoyl-L-homoserine lactone (OC12-HSL) produced by *P. aeruginosa* by suppressing its own exotoxin production while enhancing expression of protein A, a key surface virulence factor (Qazi et al., 2006). Conversely, it indicated that AHLs from *P. aeruginosa* and *A. baumannii* function reciprocally to promote biofilm formation in both species, with heterologous AHLs significantly increasing biofilm mass, surface area coverage, and biovolume, and inducing autoinducer synthase gene promoters in a cross-species manner. Notably, *A. baumannii* growth was not inhibited by *P. aeruginosa* pyocyanin, suggesting that these two pathogens can coexist and interact synergistically in polymicrobial infections (Bhargava et al., 2012). Beyond pathogen-to-pathogen interactions, AHL signaling can also be disrupted by commensal or probiotic organisms. It showed that *Lactobacillus brevis* strain 3 M004 degrades OC12-HSL and significantly inhibits *P. aeruginosa* biofilm (up to 33% inhibition) and pyocyanin production (up to 30.75% inhibition), with transcriptomic analysis revealing downregulation of *lasA*, *lasB*, and the pyocyanin biosynthesis key gene *phzAB* (Liang et al., 2022). These findings collectively illustrate that AHL-mediated signaling is not unidirectional but rather a dynamic network in which pathogens can antagonize, synergize, or be quenched by other microbial players, with profound implications for both polymicrobial pathogenesis and the development of QS-targeted therapeutic strategies. It is important to note that while *P. aeruginosa*, *A. baumannii*, and *S. aureus* are not primary oral pathogens, they are included here as well-characterized model organisms that illustrate fundamental AHL-mediated QS principles applicable to oral biofilms.

### 2.2 QS as a driver of synergistic and antagonistic community interactions

The activation and output of QS within oral biofilms are not uniform across the community; rather, they are profoundly constrained by the three-dimensional architecture of the biofilm. Mature oral biofilms are structured as densely packed microcolonies encased in an extracellular polymeric substance (EPS) matrix, interspersed with water channels and characterized by steep physiological gradients of oxygen, pH, and nutrients. Unlike the well-mixed conditions of planktonic cultures, autoinducer accumulation in biofilms is governed by local cell density, diffusional hindrance imposed by the EPS, and convective dilution in fluid channels. These physical constraints generate substantial spatial heterogeneity in signal concentration, such that the effective AI-2 or AHL levels perceived by cells in deep, nutrient-limited layers may differ markedly from those at the biofilm periphery, giving rise to localized QS activation zones and metabolically distinct subpopulations within the same multispecies consortium. This architectural complexity has dual therapeutic implications: it creates ecological refuges where persister-like cells may evade QS-dependent clearance, while simultaneously imposing a penetration barrier that QSIs must overcome to reach their targets in deeper strata. Thus, understanding the interplay between biofilm spatial organization and QS dynamics is essential for devising strategies that effectively disrupt communication within sheltered microcolonies.

Within multispecies biofilms, QS orchestrates a dynamic balance of cooperation and conflict. Synergistic interactions are exemplified by *F. nucleatum* bridging early colonizers, including *streptococci* and *actinomyces*, with late anaerobes via AI-2-dependent coaggregation (Jang et al., 2013). Another striking example is metabolic crosstalk: ornithine excreted by *S. gordonii* stimulates polyamine synthesis in *F. nucleatum*, enhancing methionine pathway activity and elevating methyl mercaptan (CH3SH) production, which is a major halitosis and periodontitis mediator (Hara et al., 2024). *P. gingivalis* and *T. denticola* can antagonize *S. mutans* CSP-mediated virulence, potentially reducing cariogenic activity at sites where periodontal pathogens dominate (Wang et al., 2011).

Antagonistic interactions are equally common. AI-2 from *V. tobetsuensis* inhibits *S. gordonii* biofilm, and *Streptococcus cristatus* (*S. cristatus*) surface arginine deiminase (ArcA) suppresses *P. gingivalis* growth via QS interference (Mashima and Nakazawa, 2017; Xie et al., 2007). Lactobacillus species downregulate *S. mutans* QS genes including *vicKR* and *comCD* and reduce exopolysaccharide synthesis, offering a probiotic mechanism against caries (Wasfi et al., 2018). In the Rgg/SHP system, *S. mutans* tryglysins specifically inhibit competing *streptococci* such as *S. sanguinis* but not *E. faecalis*, suggesting niche-specific targeting (Brown et al., 2015).

Cross-kingdom interactions add further complexity. The polymorphic fungus *Candida albicans* (*C. albicans*) regulates its own morphogenesis and virulence through at least two QS molecules, farnesol and tyrosol, which coordinate the yeast-to-hypha transition and biofilm formation (De Sordi and Mühlschlegel, 2009; Han et al., 2011). Farnesol also serves as a key mediator of bacterial-fungal interactions. AI-2 from *A. actinomycetemcomitans* inhibits *C. albicans* hyphal and biofilm formation, while *C. albicans* farnesol suppresses *Pseudomonas* quinolone signal (PQS) production (Krom et al., 2014). Beyond *Pseudomonas*, Vila et al. indicated that farnesol modulates *S. aureus* pathogenesis by inducing depigmentation through interference with staphyloxanthin biosynthesis, likely via competitive binding to the CrtM enzyme, and activates the thiol-based oxidative stress response, paradoxically conferring enhanced tolerance to H₂O₂ and phagocytic killing (Vila et al., 2019). In mixed *S. mutans*-*C. albicans* biofilms, farnesol promotes *S. mutans* microcolony development by enhancing *gtfB* expression, accelerating cariogenicity (Cai and Kim, 2023). *E. faecalis* negatively impacts *C. albicans* hyphal morphogenesis partially through its Fsr QS system (Cruz et al., 2013). While the fungal QS discussion here is limited to illustrative examples (farnesol and tyrosol), these are included to provide evidence for principles of cross-kingdom dialogue that are increasingly recognized as relevant to oral biofilm ecology and therapeutic targeting. These interkingdom dialogues, mediated by molecules such as farnesol and AI-2, have profound implications for biofilm architecture, polymicrobial pathogenesis, and disease severity (Supplementary Table S1).

### 2.3 Pathogenic significance of QS in disease models and dysbiosis

**Figure 1:**
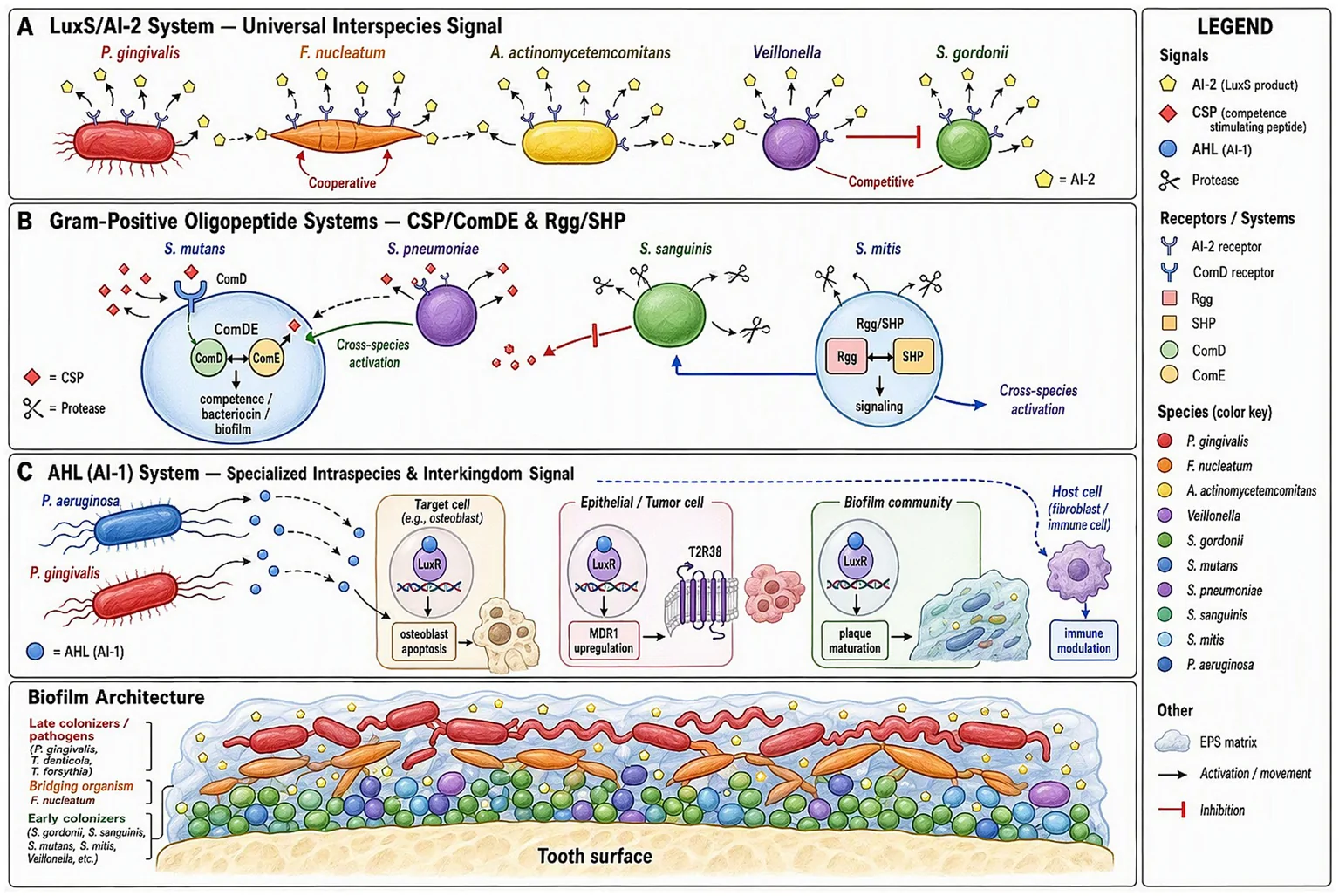
Schematic overview of quorum sensing circuits in oral multispecies biofilms. Three QS systems operate in oral biofilms: **(A)** LuxS/AI-2 mediates interspecies signaling; **(B)** Gram-positive oligopeptides (CSP/Rgg) regulate competence; **(C)** AHL/AI-1 enables specialized intraspecies and interkingdom communication. Bottom biofilm shows early colonizers (green), *F. nucleatum* (orange), and red complex (red). Arrows indicate signal production, detection, and regulation; T-bars denote inhibition.

Beyond *in vitro* observations, the pathogenic role of QS is supported by animal models. *F. nucleatum* AI-2 promotes osteoclastogenesis and bone loss in a dose-dependent manner (Li et al., 2024). Beyond its direct effect on osteoclast differentiation, AI-2 also modulates host immune responses by promoting macrophage classical polarization through NF-κB activation, thereby exacerbating periodontal inflammation and alveolar bone resorption (Zhou et al., 2025). This pro-inflammatory effect could be reversed by the QS inhibitor D-ribose, further underscoring the therapeutic potential of QS targeting. Administration of QSIs including furanone and D-ribose in BALB/c mice co-infected with *P. gingivalis* and *F. nucleatum* significantly reduced alveolar bone loss and total bacterial burden (Ben Amara et al., 2018). In a *P. gingivalis* mono-infection model, the same inhibitors decreased bone breakdown and reduced the quantity of *P. gingivalis* DNA as measured by real-time polymerase chain reaction (Cho et al., 2016). Additionally, natural compounds such as luteolin and hypochlorous acid solution have been shown to attenuate alveolar bone loss and periodontal inflammation in rodent periodontitis models, at least partially through QS interference (Fan et al., 2026). Metatranscriptomic analyses of subgingival biofilms from periodontitis patients reveal that QS-related genes (K09936) are significantly overrepresented, and quorum-quenching (QQ) strains are more frequent in diseased sites, implying that disrupted communication may favor dysbiosis (Muras et al., 2020a; Zhang et al., 2019). In young patients with Grade C periodontitis under maintenance therapy, genes for QS and efflux pumps remained more abundant than in healthy controls, despite taxonomic shifts toward *Streptococcus* (Paz et al., 2022). Furthermore, cyclodipeptides produced by *Synergistetes*, a phylum enriched in periodontitis, inhibit commensal growth, promoting pathobiont emergence (Marchesan et al., 2015). Supporting these clinical observations, *ex vivo* treatment of subgingival plaque samples from advanced periodontitis patients with the AHL-lactonase Est816 reduced biofilm biomass by 64% and thickness by 76%, demonstrating the translational potential of QS-targeted approaches in patient-derived specimens (Zhao et al., 2025b). Collectively, these data provide evidence linking QS activity to disease initiation and progression, spanning from molecular immune modulation to animal models and clinical dysbiosis (Figure 1).

## 3 QS-targeted therapeutic strategies

Before surveying the diverse array of QSIs reported in the literature, a critical framework for evaluating their evidence is warranted. Throughout this section, we categorize reported QSIs according to the strength of evidence supporting their purported mechanism of action. Class I agents are those for which QS inhibition has been rigorously demonstrated through multiple orthogonal assays, including direct measurement of signal reduction, receptor-binding assays, and demonstration of density-dependent phenotype reversal. Class II agents exhibit antibiofilm or anti-virulence activity that correlates with reduced QS gene expression but lacks direct evidence of signal interference, leaving open the possibility of indirect or pleiotropic mechanisms. Class III agents are reported as QSIs based solely on phenotypic assays (e.g., reduced biofilm biomass or pigment production) without mechanistic validation, and their activity may equally reflect general antimicrobial effects, membrane disruption, or metabolic inhibition. This classification is not intended to diminish the value of promising leads but to encourage interpretative caution and to highlight priorities for mechanistic follow-up studies.

### 3.1 Natural product-derived QSIs

A wealth of natural compounds exhibit QS-inhibitory activity. Green tea epigallocatechin-3-gallate (EGCG) synergizes with antibiotics against *P. gingivalis*, reduces epithelial adherence, and downregulates colonization and tissue destruction genes via AI-2 interference (Fournier-Larente et al., 2016). Catechins containing pyrogallol-type B-rings and/or galloyl groups inhibit *E. corrodens* biofilm by disrupting AI-2 signaling (Matsunaga et al., 2010). Coumarin, a plant-derived benzopyrone, suppresses *P. gingivalis* growth, disrupts biofilm architecture, downregulates biofilm-related genes, and, as revealed by molecular docking, interferes with LuxS/AI-2 activity and HmuY heme-binding (He et al., 2022). Commiphora extracts exhibit anti-QS and antimicrobial activity against *Moraxella catarrhalis* (*M. catarrhalis*) and *S. aureus* (Rubegeta et al., 2019). 24-propylcholesterol from *Piper betel* Linn inhibits *E. faecalis* virulence factors and QS (Windaryanti et al., 2022). Umbelliferon derivatives from *Ferula narthex*, particularly Feselol, show robust antibiofilm activity against *P. aeruginosa* and *staphylococci* from diabetic periodontitis patients (Amin et al., 2020). Essential oils from *Thymus linearis*, *Salvia officinalis*, *Satureja hortensis*, and *Anethum graveolens* exhibit anti-QS, antibiofilm, and immunomodulatory properties in oral cell models (Rafey et al., 2021; Popa et al., 2020).

Recent studies have expanded this repertoire with novel mechanisms and sources. Gum Arabic interferes with AI-2 signaling in key periodontal pathogens while promoting beneficial bacteria and enhancing immune responses (Hashim et al., 2024). Phloretin and phlorizin significantly reduced mixed-species biofilm formation and decreased AI-2 activity to 45.9 and 55.4%, respectively (Wu et al., 2024). Ferulic acid suppresses *P. gingivalis* biofilm through dual inhibition of AI-2 production and receptor activity (Ando et al., 2025). Fermented ginger extract, via bioconversion of 6-gingerol to 6-shogaol, achieves 60–70% biofilm inhibition and downregulates *luxS* as well as adhesion-associated genes including *flp*, *fimA*, *fadA*, *ltxA*, and *rgpB* (Li et al., 2026). Asiatic acid disrupts *S. mutans* biofilm (minimum biofilm inhibitory concentration: 62.5 μM) through transcriptional reprogramming of the CiaRH system (Shi et al., 2025). Thymoquinone inhibits biofilm formation and virulence of *F. nucleatum* and *P. gingivalis* (Ahmad et al., 2025). Collectively, these emerging natural products offer diverse and promising strategies for QS-targeted periodontal therapy.

While the natural products enumerated above present an impressive array of antibiofilm activity, a critical caveat must be acknowledged: the majority of these studies have not rigorously distinguished QS inhibition from other mechanisms of action. Many plant-derived polyphenols and flavonoids, including EGCG, curcumin, and coumarin, are known to possess broad-spectrum antimicrobial, antioxidant, and membrane-disrupting properties. Reduced biofilm formation or downregulated QS gene expression following treatment may therefore reflect generalized stress responses, metabolic inhibition, or cytotoxicity rather than specific disruption of QS circuitry. Moreover, the concentrations at which these compounds exhibit activity *in vitro* are often substantially higher than those that would be achievable *in vivo* following topical or systemic administration, raising questions about clinical feasibility. Future studies should prioritize: (i) structure–activity relationship analyses to identify the minimal pharmacophore responsible for QS inhibition; (ii) demonstration of QS-specific effects using receptor-binding assays, signal quantification, and density-dependence experiments; and (iii) evaluation of activity at physiologically relevant concentrations using relevant biological matrices such as saliva or gingival crevicular fluid. Without such validation, the attribution of antibiofilm activity to QS inhibition in natural products remains largely suggestive rather than definitive.

### 3.2 Synthetic small molecules and analogues

Furanone derivatives are among the best-characterized synthetic QSIs. (5Z)-4-bromo-5-(bromomethylene)-2(5H)-furanone inhibits single- and dual-species biofilm formation between *F. nucleatum* and red-complex pathogens, downregulating FadA, RgpA, Msp, and BspA (Jang et al., 2013). Novel bromofuranone analogs, specifically 3-(dibromomethylene) isobenzofuran-1(3H)-one derivatives, show potent anti-biofilm activity against *F. nucleatum*, *P. gingivalis*, and *T. forsythia* (Park et al., 2017). Monosaccharide competitors exploit AI-2 recognition: D-ribose inhibits AI-2 activity in triple-species biofilms containing *F. nucleatum*, *P. gingivalis*, and *A. actinomycetemcomitans* without affecting planktonic growth (Li et al., 2024). D-arabinose and D-galactose inhibit biofilm formation on titanium discs and, intriguingly, generate outer membrane vesicles (OMVs) with reduced proinflammatory activity (An et al., 2022, 2023).

Curcumin-based formulations have also shown promise. Curcumin-decorated nanophytosomes combined with photo-sonodynamic therapy (PSACT) reduce *A. actinomycetemcomitans* viability, degrade biofilms, and downregulate QS-associated *qseB* and *qseC* as well as biofilm-related *rcpA* (Pourhajibagher and Bahador, 2021). Curcumin-mediated antimicrobial photodynamic therapy (Cur-aPDT) similarly reduces metabolic activity, efflux capacity, and QS ability (Mahdizade-Ari et al., 2019). Other synthetic agents have been investigated. 4-hydroxycinnamic acid inhibits *S. mutans* biofilm and QS (Ramsundar et al., 2023b). The corticosteroid derivative PYED-1 downregulates QS, surface protein, and toxin genes in *S. aureus* (Vollaro et al., 2020). Organosulfur compounds target AI-2 to inhibit multispecies biofilm development (Kasper et al., 2014). Short-chain fatty acids including acetate, propionate, and butyrate downregulate CSP system genes in *S. gordonii* (Park et al., 2021). Sulfated vizantin promotes detachment of *Streptococcus*-dominated biofilms without bactericidal effects, thereby preserving resident flora (Hasegawa et al., 2020). Baicalein downregulates *luxS*, *comDE*, *comA*, and *comB* in *S. mutans*, inhibiting biofilm without affecting *S. gordonii* (Vijayakumar et al., 2021). Importantly, CSP analogs from *S. pneumoniae* can antagonize *S. mutans* ComDE, reducing biofilm and protecting commensal streptococci (Bernabè et al., 2022).

Recent studies have expanded the synthetic QSI arsenal with novel compounds and strategies. D-histidine, a non-proteinogenic amino acid, inhibits *A. actinomycetemcomitans* biofilm formation and disrupts established biofilms in a concentration-dependent manner without affecting bacterial growth, while downregulating QS-related virulence genes; notably, it synergizes with antibiotics including amoxicillin, minocycline, and metronidazole to enhance biofilm eradication (Shan et al., 2025). Hypochlorous acid solution targets QS of periodontal pathogens by inhibiting AI-2 activity and downregulating QS-associated gene expression, offering an antibiofilm approach with favorable *in vitro* biocompatibility; however, its *in vivo* safety profile and long-term biocompatibility require further investigation (Lv et al., 2025). Antimicrobial peptide-targeted photodynamic therapy using nanocomposites significantly downregulates the LuxS/AI-2 QS system, reducing AI-2 synthesis and virulence gene expression (Li et al., 2025). Peptide-based approaches have also emerged, with the QS peptide analogue KBI-3221 decreasing biofilm formation of various *Streptococcus* species, and peptide-coumarin conjugates showing promise in disrupting QS and biofilm formation (Khan et al., 2026).

### 3.3 Enzymatic quorum quenching: AHL-lactonases

Rather than inhibiting signal synthesis or reception, enzymatic degradation of AHLs offers a catalytic and potentially long-lasting strategy. AHL-lactonase SsoPox and GcL increase the relative abundance of commensal Gram-positive taxa including *Lactobacillales*, *Actinomyces*, and *Streptococcus* in oral biofilms (Sikdar et al., 2025). Aii20J, an AHL-lactonase, significantly inhibits biofilm formation from periodontitis patient saliva, disrupts established mixed-species biofilms, and increases *Streptococcus oralis* (*S. oralis*) subsp. *dentisani* while decreasing *Streptococcus vestibularis* (*S. vestibularis*), representing a shift toward a less cariogenic and periodontopathic profile. Notably, Aii20J also enriches arginine deiminase- and urease-producing species including *S. cristatus* and *Streptococcus salivarius* (*S. salivarius*), which buffer acidic pH (Parga et al., 2023b; Parga et al., 2023a).

**Figure 2:**
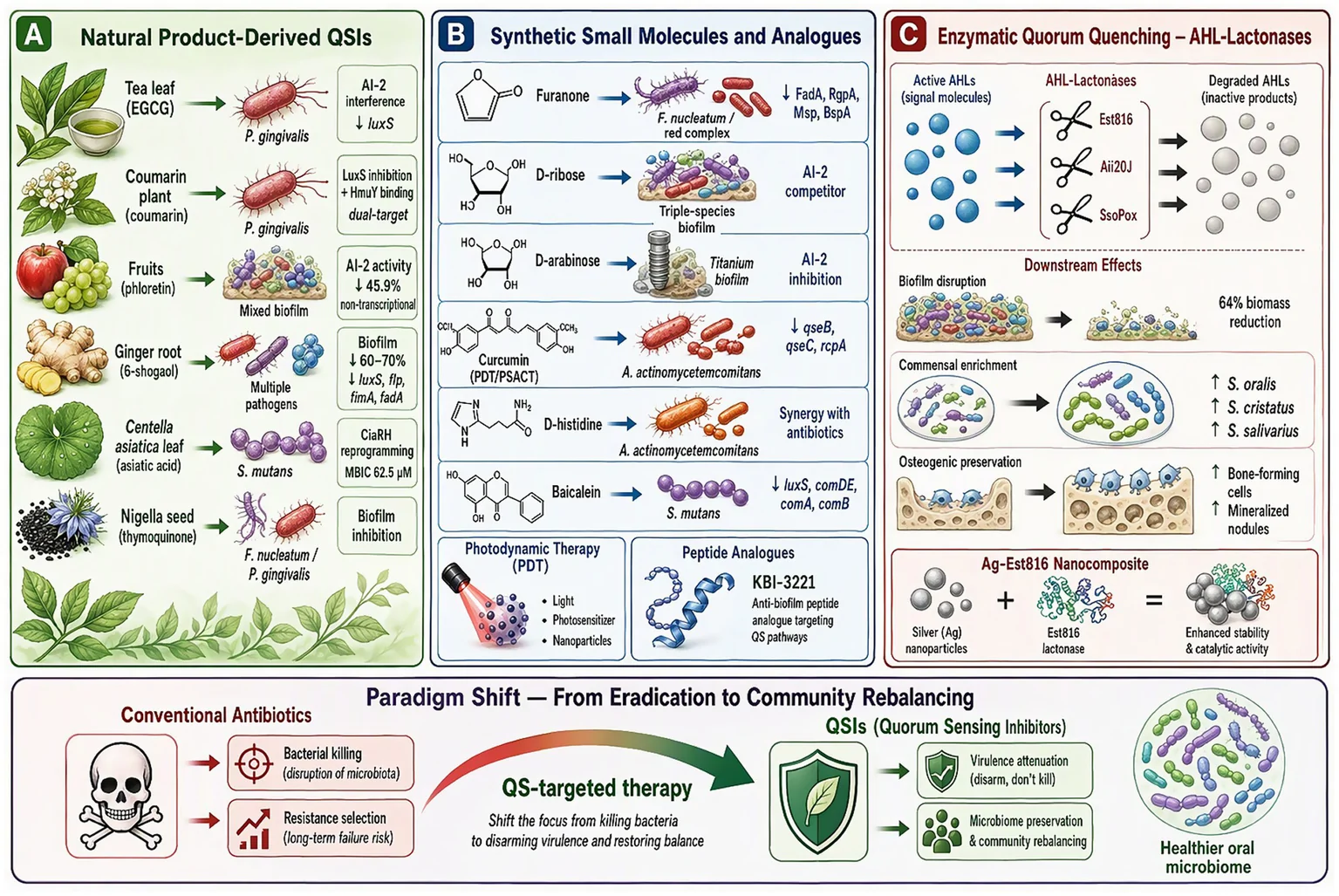
Quorum sensing inhibitor strategies targeting oral biofilms. Three QSI categories: **(A)** Natural products (EGCG, coumarin, phloretin, 6-shogaol, asiatic acid, thymoquinone) targeting AI-2, receptors, or transcription; **(B)** Synthetic molecules (furanones, D-ribose, D-arabinose, curcumin, D-histidine, baicalein) inhibiting biofilms and virulence, with PDT/peptide approaches; **(C)** AHL-lactonases (Est816, Aii20J, SsoPox) degrading AHLs to disrupt biofilms, enrich commensals, and preserve osteogenesis. Bottom panel contrasts antibiotics (killing) with QSIs (disarming), highlighting community rebalancing.

Est816, another lactonase, inhibits *A. actinomycetemcomitans* biofilm and virulence, reduces leukotoxin (*ltxA*) and fimbrial (*rcpA*) expression, and synergizes with minocycline to alleviate periodontitis in rats, reducing bone resorption and inflammatory damage (Wang et al., 2024; Zhao et al., 2024). It also disrupts *P. gingivalis* biofilms, suppresses virulence genes including *rgpA*, *kgp*, and *fimA*, and attenuates pro-inflammatory cytokine secretion while preserving osteogenic differentiation of periodontal ligament stem cells (Zhao et al., 2025a). In patient-derived subgingival plaque samples, Est816 reduces biofilm biomass and thickness (Zhao et al., 2025b). A silver nanocomposite incorporating Est816 (Ag-Est816) has further been developed, demonstrating potent antibiofilm activity with non-cytotoxicity to human gingival fibroblasts, collectively positioning Est816 as a promising adjunctive therapy for periodontitis management (Zhao et al., 2026) (Supplementary Table S2; Figure 2).

## 4 Challenges and future perspectives

Despite the substantial preclinical evidence establishing QS as a druggable target in oral biofilms, no QSI has yet entered clinical use for periodontal or cariogenic diseases, and several critical barriers must be addressed to bridge the translational gap. It must be acknowledged, however, that QS-targeted therapy remains fundamentally a preclinical concept with respect to clinical application. While the mechanistic rationale is robust and the preclinical antibiofilm data are compelling, the leap from *in vitro* efficacy and animal models to human periodontal or cariogenic disease is substantial. Unlike conventional antibiotics, which have well-established pharmacodynamic targets (minimum inhibitory concentrations, time-kill curves) and decades of clinical validation, QSIs lack validated surrogate endpoints, standardized dosing regimens, and any demonstration of clinically meaningful benefit in human subjects. This evidentiary gap should temper expectations and frame the discussion that follows not as a near-term therapeutic reality, but as a long-term research agenda requiring systematic, milestone-driven progress. Many natural QSIs, such as EGCG and curcumin, exhibit pleiotropic bioactivities that impact host signaling pathways and microbial metabolism beyond their intended QS targets, complicating mechanistic attribution and raising safety concerns; achieving selective targeting through receptor-specific antagonists, signal-degrading enzymes with narrow substrate preferences, or rationally designed peptide analogues is therefore essential to avoid unintended dysbiosis. Recent findings that *F. nucleatum* AI-2 production is restricted to a single subspecies and uncoupled from QS further underscore the need for precise targeting, as inhibitors designed against a signal that is not universally produced may have limited efficacy in heterogeneous clinical populations.

The oral environment, characterized by constant salivary flow, masticatory abrasion, and rapid dilution, poses substantial challenges for drug retention, and most QSIs are not formulated for sustained release, underscoring the urgent need for advanced delivery platforms including nanoparticles, hydrogels, mucoadhesive films, or chewing gum-based formulations. Beyond physical barriers of dilution and clearance, a fundamental translational gap lies in the uncertainty surrounding physiologically relevant QS signal concentrations. The majority of preclinical studies have employed exogenous AI-2, AHLs, or synthetic analogues at supraphysiological concentrations (often in the micromolar to millimolar range), yet the actual concentrations of native autoinducers in subgingival crevicular fluid, saliva, or plaque fluid remain poorly characterized, with available measurements suggesting substantial inter-individual variability and site-specific heterogeneity. This disparity complicates the interpretation of QSI efficacy, as inhibitors that effectively block communication at artificially high signal concentrations may prove insufficient in the low-signal, diffusion-limited microenvironment of deep periodontal pockets. Moreover, the oral cavity is enzymatically active: host-derived salivary acylases and lactonases, along with microbial degradation pathways (e.g., the AI-2-inactivating enzyme identified in *E. corrodens*), can rapidly turn over signaling molecules, further shortening their functional half-life and reducing the effective concentration available for QS activation or inhibition. These factors collectively underscore the urgent need for quantitative pharmacokinetic and pharmacodynamic studies that measure autoinducer concentrations *in situ* and evaluate QSI activity under physiologically relevant conditions including the use of human saliva or gingival crevicular fluid as culture media rather than relying solely on conventional nutrient-rich laboratory media.

Compounds such as furanones and brominated derivatives may exhibit cytotoxicity to epithelial or immune cells at effective concentrations, and while enzymes like Est816 and Aii20J have shown favorable safety profiles *in vitro*, systematic biocompatibility assessments in 3D oral mucosal models and long-term animal studies remain conspicuously limited. Although QSIs are often described as imposing lower selective pressure than conventional bactericides since they target communication rather than viability, this does not render them immune to resistance evolution. Bacterial populations can acquire resistance through multiple mechanisms, including: (i) mutations in signal receptors that reduce inhibitor binding while preserving native signal recognition; (ii) overproduction of autoinducers to outcompete inhibitors; (iii) redundancy among parallel QS pathways that compensate for the inhibited circuit; and (iv) upregulation of efflux pumps that actively eliminate QSIs from the cell. Importantly, it is plausible, though not yet empirically demonstrated in oral pathogens, that the fitness cost of QSI resistance could be lower than that of antibiotic resistance, because QS-targeted compounds do not directly impair growth. The redundancy of AI-2 sources in complex oral communities, as indicated by compensatory AI-2 from resident microbiota sustaining *A. actinomycetemcomitans* colonization in primate models, highlights a particularly challenging scenario for AI-2-targeted therapies. To date, no longitudinal resistance surveillance has been conducted for oral QSIs, representing a critical knowledge gap that must be addressed through both *in vitro* evolution experiments and *in vivo* monitoring before clinical translation can be safely pursued.

The synergistic potential of QSIs with antibiotics, as suggested for Est816 combined with minocycline and D-histidine with amoxicillin, is encouraging; however, optimal dosing, timing, and sequence of administration remain undefined and require systematic pharmacokinetic and pharmacodynamic modeling.

A fundamental but often unacknowledged distinction between QSIs and conventional antibiotics lies in the evidence standards required for regulatory approval and clinical adoption. For antibiotics, the path to clinical use is well-trodden: *in vitro* susceptibility testing (MIC/MBC), preclinical toxicity and pharmacokinetic profiling, and Phase I-III trials with well-defined endpoints such as clinical cure rates, microbiological eradication, and prevention of resistance emergence. For QSIs, however, the evidentiary requirements are both different and, in some respects, more demanding. First, because QSIs are anti-virulence rather than bactericidal agents, traditional endpoints (e.g., reduction in bacterial load) may not capture their therapeutic benefit, necessitating the validation of novel surrogate endpoints such as reduction in inflammatory biomarkers, shifts in microbial community composition, or attenuation of specific virulence factors. Second, the absence of a direct growth-inhibitory effect means that standard susceptibility testing is irrelevant, and new assays must be developed to quantify QSI potency in a clinically meaningful way, assays that correlate with patient outcomes rather than simply with AI-2 activity *in vitro*. Third, the long-term safety profile of QSIs must be established not only with respect to host toxicity but also with respect to ecological consequences: chronic modulation of microbial communication could have unpredictable effects on the commensal microbiome and host immunity, effects that may not manifest in short-term animal studies. Finally, regulatory frameworks for anti-virulence agents are still evolving; no dedicated guidance exists from agencies such as the FDA or EMA for the approval of QSIs, creating uncertainty about the clinical trial design and endpoints that would be acceptable (Czaplewski et al., 2016; Theuretzbacher et al., 2019). These heightened evidentiary demands underscore the need for a deliberate, phased approach to clinical translation, beginning with rigorous proof-of-principle studies in well-characterized patient populations before any claims of therapeutic efficacy can be advanced.

Moreover, preclinical studies currently employ disparate outcome measures including biofilm biomass, metabolic activity, viability, gene expression, and alveolar bone loss which severely hinders cross-study comparison. To address this, we propose a tiered framework of standardized endpoints for future QSI evaluations. First, clinical parameters should be prioritized, including reductions in probing depth, gains in clinical attachment level, and improvements in bleeding on probing, as these are the gold-standard metrics for periodontal disease. Second, microbiological endpoints should incorporate quantitative assessments of community composition via 16S rRNA or metagenomic sequencing, shifts in the pathogen-to-commensal ratio (e.g., red-complex species vs. health-associated taxa), and expression profiling of QS-regulated virulence genes. Third, QS-specific biomarkers such as direct quantification of AI-2 or AHL concentrations in subgingival plaque or crevicular fluid using reporter bioassays or mass spectrometry, alongside functional assays of protease, hemagglutination, or exopolysaccharide production would provide mechanistic confirmation of target engagement. These endpoints should be evaluated longitudinally and, where possible, correlated with one another to establish clinically meaningful thresholds for therapeutic efficacy. Achieving consensus on such a framework is urgently needed to standardize future evaluations and enable meaningful cross-study comparisons. Finally, an underexplored frontier lies in the interplay between QS and antimicrobial persistence; in non-oral pathogens, QS upregulates efflux pumps and stress responses, thereby contributing to persister cell formation, yet whether analogous mechanisms operate in *P. gingivalis* or *S. mutans* biofilms remains entirely unknown, and elucidating this connection could rationally guide the design of QSI-antibiotic combination regimens to eradicate recalcitrant oral infections.

Complementary to the Class I-III framework introduced in Section 3 for categorizing individual QSI agents, we propose a provisional evidence-grading framework to guide the interpretation of QSI studies as a whole. Grade A evidence requires demonstration of: (i) signal reduction or receptor interference (direct biochemical evidence); (ii) density-dependent phenotype reversal; (iii) restoration of phenotype by exogenous signal; and (iv) correlation between QSI potency and therapeutic efficacy in a relevant *in vivo* model. To date, only AHL-lactonases such as Est816 and Aii20J, and select synthetic AI-2 antagonists such as D-ribose and certain furanone derivatives, meet some but not all of these criteria. Grade B evidence includes studies demonstrating QS gene downregulation or phenotypic attenuation in biofilm models, but lacking direct biochemical confirmation of signal interference, this category encompasses the majority of natural product studies. Grade C evidence comprises studies reporting antibiofilm activity that is assumed to be QS-mediated based solely on phenotypic readouts, with no mechanistic investigation. Adopting such a framework would sharpen the focus of future research efforts toward compounds with validated mechanisms and away from the continued accumulation of uncharacterized, potentially pleiotropic inhibitors. It would also facilitate cross-study comparisons and accelerate the identification of truly QS-specific agents worthy of clinical development.

Looking forward, several promising directions merit prioritization: the development of highly specific receptor-blockers or signal-degrading enzymes with engineered substrate selectivity informed by structural biology; the integration of QSIs into sustained-release delivery systems tailored to the oral cavity; rigorous longitudinal resistance surveillance employing both *in vitro* evolution experiments and *in vivo* monitoring; multi-modal strategies that combine QS modulation with photodynamic therapy, antimicrobial peptides, or biofilm-dispersing agents; and, given the subspecies-specific heterogeneity uncovered in *F. nucleatum* and other oral taxa, patient- or site-specific microbiome profiling to inform precision therapeutic approaches rather than one-size-fits-all strategies.

## 5 Conclusion

QS remains primarily a preclinical concept with respect to therapeutic application. This review consolidates evidence positioning QS as a central regulatory hub in oral biofilm assembly and pathogenesis, with the LuxS/AI-2, oligopeptide, and AHL systems collectively contributing to the regulation of polymicrobial virulence and microbial interactions. Despite potent antibiofilm effects of natural, synthetic, and enzymatic QSIs in preclinical models, clinical translation is obstructed by formulation challenges, safety uncertainties, and a lack of standardized protocols, necessitating future efforts in precision targeting, sustained delivery, resistance surveillance, and combination regimens to realize the paradigm shift from microbial eradication to community rebalancing.
