# Supplementary material for: Quorum sensing in oral multispecies biofilms: from interkingdom communication to therapeutic targeting
Source: Front Microbiol. 2026 Sep 7;17:1957601. doi: 10.3389/fmicb.2026.1957601 (PMC13591311; doi:10.3389/fmicb.2026.1957601)
Supplement: Supplementary file 1 [file Supplementary_file_1.DOCX]

**Table S1.** Key quorum sensing systems and functions in oral pathogens

| **Species** | **QS System** | **Key Functions** | **Author, year** |
| --- | --- | --- | --- |
| *P. gingivalis* | LuxS/AI-2 | Reported to regulate cysteine proteases Rgp and Kgp for tissue invasion and immune evasion | Frias et al., 2001  Burgess et al., 2002 |
|  |  | Modulates heme acquisition via HmuY/HmuR system through LuxR-family regulator PG1237 | Wu et al., 2009 |
|  |  | Observed to regulate host inflammatory responses via outer membrane protein PGN_0482; *luxS* and *PGN_0482* mutants showed impaired induction of IL-1β, IL-6, and MCP-1 | Scheres et al., 2015 |
|  | AHL/AI-1 | Produces trace amounts of OC8-HSL; LuxR-family regulator PG_2094 upregulated in biofilms; PG1237 regulates heme acquisition | Muras et al., 2020a Sánchez et al., 2019  Wu et al., 2009 |
| *F. nucleatum* | LuxS/AI-2 | Upregulates FadA adhesion and red-complex adhesion molecules (RgpA, Msp, BspA), promoting coaggregation | Jang et al., 2013 |
|  |  | Reported to promote RANKL-induced osteoclast differentiation and alveolar bone loss in murine models | Li et al., 2024 |
|  |  | AI-2 production restricted to FNA subspecies; FNN and FNV lack *luxS*, FNP carries disrupted homolog; AI-2 uncoupled from QS | G C et al., 2026 |
| *A. actinomycetemcomitans* | LuxS/AI-2 | AI-2 upregulates leukotoxin (3-fold) and iron transport gene *afuA* (8-fold) | Fong et al., 2001 |
|  |  | RbsB identified as AI-2 receptor; *rbsB* mutant shows reduced growth under iron limitation | James et al., 2006 |
|  |  | LuxS and ArcB coordinately regulate iron transport (*afuA*, *fecBCDE*, *ftnAB*) under aerobic iron limitation | Fong et al., 2003 |
|  |  | AI-2 reduces dual-species biofilm with *C. albicans* and *S. mutans* | Bachtiar & Bachtiar, 2020 |
|  |  | Inactivation of RbsB and LsrB nearly abolishes biofilm; QseBC links signal detection to biofilm maturation | Shao et al., 2007  Novak et al., 2010 |
| *S. mutans* | CSP/ComDE | Regulates bacteriocin production, acid tolerance, competence, and biofilm formation; high CSP induces altruistic cell death | Dufour & Lévesque, 2013 |
|  |  | CSP cross-species activation by *S. pneumoniae*; inactivation by proteases from *S. sanguinis* and *S. mitis* | Bernabè et al., 2022  Ricomini Filho et al., 2019 |
| *S. mitis* | Rgg/SHP | Cross-activates *S. pneumoniae* Rgg/SHP system, modulating surface polysaccharide synthesis | Junges et al., 2019 |
| *S. pneumoniae* | CSP/ComDE | CSP can cross-activate *S. mutans* ComDE system, inducing biofilm and bacteriocin production | Bernabè et al., 2022 |
| *S. cristatus* | ArcA | Surface arginine deiminase suppresses *P. gingivalis* growth via QS interference | Xie et al., 2007 |
| *E. corrodens* | LuxS/AI-2 | AI-2 peaks in mid-log phase; LuxS facilitates biofilm maturation and detachment | Azakami et al., 2006  Karim et al., 2013a, 2013b |
| *E. faecalis* | LuxS/AI-2 | *luxS* deletion increases biofilm formation and cell-surface hydrophobicity (negative regulation) | He et al., 2016  Yang et al., 2018 |
| *C. ochracea* | LuxS/AI-2 | LuxS deficiency reduces biofilm formation | Hosohama-Saito et al., 2016 |
| *V. tobetsuensis* | AI-2-like  PPAI-2 | Inhibits *S. gordonii* biofilm formation (antagonistic interference) | Mashima & Nakazawa, 2017 |
| *S. aureus* | AHL/AI-1 | OC12-HSL from *P. aeruginosa* suppresses exotoxin production, enhances protein A expression | Qazi et al., 2006 |
| *P. aeruginosa* | AHL/AI-1 | C12-HSL modulates osteoblast apoptosis/differentiation; activates T2R38 → MDR1 upregulation | Guo et al., 2020  Gaida et al., 2016 |
| *A. baumannii* | AHL/AI-1 | Reciprocal AHL signaling with *P. aeruginosa* promotes biofilm formation; not inhibited by pyocyanin | Bhargava et al., 2012 |

**Table S2.** Quorum sensing inhibitors (QSIs) and their mechanisms

| **Category** | **Agent / Compound** | **Target Pathogen(s)** | **Mechanism of Action** | **Author, year** |
| --- | --- | --- | --- | --- |
| **Natural Products** | EGCG (green tea) | *P. gingivalis* | Suggested AI-2 interference; synergizes with antibiotics | Fournier-Larente et al., 2016 |
|  | Catechins | *E. corrodens* | Reported to disrupt AI-2 signaling | Matsunaga et al., 2010 |
|  | Coumarin | *P. gingivalis* | Suggested LuxS/AI-2 inhibition; HmuY binding | He et al., 2022 |
|  | Commiphora extracts | *M. catarrhalis, S. aureus* | Anti-QS and antimicrobial activity | Rubegeta et al., 2019 |
|  | 24-propylcholesterol (Piper betel) | *E. faecalis* | Inhibits virulence factors and QS | Windaryanti et al., 2022 |
|  | Umbelliferon derivatives (Feselol) | *P. aeruginosa, staphylococci* | Antibiofilm activity against diabetic periodontitis isolates | Amin et al., 2020 |
|  | Gum Arabic | *P. gingivalis*  *F. nucleatum*  *A. actinomycetemcomitans* | Reported AI-2 interference; prebiotic effects | Hashim et al., 2024 |
|  | Phloretin  /Phlorizin | *P. gingivalis/*  Mixed-species biofilms | AI-2 activity reduced to 45.9% / 55.4% | Wu et al., 2024 |
|  | Ferulic acid | *P. gingivalis* | Reported dual inhibition of AI-2 production and receptor activity | Ando et al., 2025 |
|  | Fermented ginger  /6-shogaol | *P. gingivalis*  *F. nucleatum*  *A. actinomycetemcomitans* | 60–70% biofilm inhibition; downregulates *luxS*, *flp*, *fimA*, *fadA*, *ltxA*, *rgpB* | Li et al., 2026 |
|  | Asiatic acid | *S. mutans* | Transcriptional reprogramming of CiaRH system; MBIC 62.5 μM | Shi et al., 2025 |
|  | Thymoquinone | *P. Gingivalis*  *F. nucleatum* | Reported to Inhibit biofilm formation and virulence | Ahmad et al., 2025 |
|  | Essential oils  (*T. linearis*, *S. officinalis*, etc.) | Oral pathogens | Anti-QS, antibiofilm, immunomodulatory | Rafey et al., 2021  Popa et al., 2020 |
| **Synthetic Small Molecules** | Furanone derivatives | *F. nucleatum*  Red-complex pathogens | Downregulates FadA, RgpA, Msp, BspA | Jang et al., 2013 |
|  | Bromofuranone analogs | *P. gingivalis*  *F. nucleatum*  *T. forsythia* | Potent AI-2 QS inhibition | Park et al., 2017 |
|  | D-ribose | *P. gingivalis*  *F. nucleatum*  *A. actinomycetemcomitans* | AI-2 activity inhibition; no effect on planktonic growth | Li et al., 2024 |
|  | D-arabinose  D-galactose | Periodontal pathogens | Biofilm inhibition on titanium; OMVs with reduced proinflammatory activity | An et al., 2022, 2023 |
|  | Curcumin nanophytosomes (PSACT) | *A. actinomycetemcomitans* | Downregulates *qseB*, *qseC*, *rcpA* | Pourhajibagher & Bahador, 2021 |
|  | Curcumin  (Cur-aPDT) | *A. actinomycetemcomitans* | Reduces metabolic activity, efflux capacity, QS ability | Mahdizade-Ari et al., 2019 |
|  | 4-hydroxycinnamic acid | *S. mutans* | Inhibits biofilm and QS | Ramsundar et al., 2023b |
|  | PYED-1 | *S. aureus* | Downregulates QS, surface protein, and toxin genes | Vollaro et al., 2020 |
|  | Organosulfur compounds | Oral pathogens | Target AI-2; inhibit multispecies biofilm development | Kasper et al., 2014 |
|  | Short-chain fatty acids | *S. gordonii* | Downregulate CSP system genes | Park et al., 2021 |
|  | Sulfated vizantin | *Streptococcus-*dominated biofilms | Promotes biofilm detachment without bactericidal effects | Hasegawa et al., 2020 |
|  | D-histidine | *A. actinomycetemcomitans* | Synergizes with amoxicillin, minocycline, metronidazole | Shan et al., 2025 |
|  | Hypochlorous acid | Periodontal pathogens | Reported AI-2 inhibition; downregulates QS genes | Lv et al., 2025 |
|  | Antimicrobial peptide-PDT | Periodontal pathogens | Reported to downregulate LuxS/AI-2 system | Li et al., 2025 |
|  | KBI-3221 (peptide analogue) | *Streptococcus* spp. | Reported to decrease biofilm formation | Khan et al., 2026 |
|  | Baicalein | *S. mutans* | Downregulates *luxS*, *comDE*, *comA*, *comB* | Vijayakumar et al., 2021 |
|  | CSP analogs | *S. mutans* | Reported to antagonize ComDE, reduce biofilm, protect commensals | Bernabè et al., 2022 |
| **Enzymatic QSIs** | SsoPox / GcL (AHL-lactonases) | Oral biofilms | Increase commensal Gram-positive taxa | Sikdar et al., 2025 |
|  | Aii20J (AHL-lactonase) | Periodontitis patient biofilms | Inhibits biofilm; enriches pH-buffering species (*S. cristatus*, *S. salivarius*) | Parga et al., 2023a, 2023b |
|  | Est816 (AHL-lactonase) | *P. gingivalis A.actinomycetemcomitans* | Suppresses *ltxA*, *rcpA*, *rgpA*, *kgp*, *fimA*; synergizes with minocycline; reduces bone resorption | Wang et al., 2024  Zhao et al., 2024  Zhao et al., 2025 |
|  | Ag-Est816 nanocomposite | *P. gingivalis*  *S. sanguinis* | Disrupts dual-species biofilms; non-cytotoxic to gingival fibroblasts | Zhao et al., 2026 |
